# Clozapine & constipation: an audit of bowel habit monitoring and laxative prescribing in inpatients on clozapine

**DOI:** 10.1192/bjo.2021.220

**Published:** 2021-06-18

**Authors:** Holly Boyd, Anna Manso de Zuniga

**Affiliations:** Merseycare NHS Foundation Trust

## Abstract

**Aims:**

To establish how often bowel habits are monitored in inpatients on clozapine

To determine how many of these patients are prescribed laxatives and whether these are utilised

**Background:**

It's estimated that 30-60% of patients will suffer from constipation whilst on clozapine; this can lead to ileus, intestinal obstruction and bowel ischaemia, all of which can be fatal. Constipation is much more common than clozapine-induced blood dyscrasias, and has a higher mortality rate. Despite this, there is no strict universal framework for bowel habit monitoring equivalent to the compulsory FBC monitoring. Local trust guidance indicates that bowel habits should be monitored regularly, at least at any point of blood sampling. However, monitoring processes across the trust were noted to be variable, as were laxative prescribing practices.

**Method:**

The data sample of current inpatients on clozapine across the trust was identified from pharmacy records. The patient's Rio notes from the preceding 3 months were searched for predetermined terms relating to bowel habits and constipation, and the notes were then analysed for assessment of bowel habit. The number of FBCs collected during this 3 month period was then used to produce comparison with the audit standard. The data on laxative prescribing were collected from current medication lists on EPMA.

**Result:**

A data sample of 31 current inpatients was identified. The audit found that only 54.8% (17) of patients had their bowel habits monitored at least with every FBC taken. There was significant variability between different wards, with the best performing ward having 100% adherence to the audit standard, and the worst performing having 0%. In terms of laxative prescribing, it was found that 87.1% (27) of patients had at least 1 regular or 1 PRN laxative prescribed. Regular laxatives were prescribed for 61.2% (19) of patients, whereas only PRN laxatives were prescribed in 25.8% (8) of patients. Of those prescribed only PRN laxatives, only 50% (4) ever utilised this medication.

**Conclusion:**

Bowel habits are not consistently monitored across the trust in inpatients on clozapine, leaving room for potentially life-threatening side effects to be missed. Additionally, regular laxative prescribing is not standard throughout the trust, which could further add to the potential for significant constipation-related morbidity to occur. A standard method of monitoring bowel habits throughout the trust, as well as a trust laxative prescribing policy, could be a way of remedying this issue and preventing harmful outcomes for our patients on clozapine.

